# Simulation of a Bio-Inspired Flocking-Based Aggregation Behaviour in Swarm Robotics

**DOI:** 10.3390/biomimetics9110668

**Published:** 2024-11-01

**Authors:** Samira Rasouli, Kerstin Dautenhahn, Chrystopher L. Nehaniv

**Affiliations:** 1 Department of Electrical and Computer Engineering, University of Waterloo, Waterloo, ON N2L 3G1, Canada; kerstin.dautenhahn@uwaterloo.ca (K.D.); chrystopher.nehaniv@uwaterloo.ca (C.L.N.); 2 Department of Systems Design Engineering, University of Waterloo, Waterloo, ON N2L 3G1, Canada

**Keywords:** swarm robotics, Reynolds’ rules, aggregation behaviour, K-means

## Abstract

This paper presents a biologically inspired flocking-based aggregation behaviour of a swarm of mobile robots. Aggregation behaviour is essential to many swarm systems, such as swarm robotics systems, in order to accomplish complex tasks that are impossible for a single agent. In this work, we developed a robot controller using Reynolds’ flocking rules to coordinate the movements of multiple e-puck robots during the aggregation process. To improve aggregation behaviour among these robots and address the scalability issues in current flocking-based aggregation approaches, we proposed using a K-means algorithm to identify clusters of agents. Using the developed controller, we simulated the aggregation behaviour among the swarm of robots. Five experiments were conducted using Webots simulation software. The performance of the developed system was evaluated under a variety of environments and conditions, such as various obstacles, agent failure, different numbers of robots, and arena sizes. The results of the experiments demonstrated that the proposed algorithm is robust and scalable. Moreover, we compared our proposed algorithm with another implementation of the flocking-based self-organizing aggregation behaviour based on Reynolds’ rules in a swarm of e-puck robots. Our algorithm outperformed this method in terms of cohesion performance and aggregation completion time.

## 1. Introduction

In multi-robot systems, simple robots cooperate with each other to perform complex tasks that are often challenging or even not possible for only one robot to accomplish [1]. The use of multi-robot systems can be beneficial in several robotic applications where solving a problem requires the cooperation of multiple agents, such as search and rescue, specifically in dangerous areas, underwater and space exploration, autonomous vehicles, and so forth. In these application areas, several simple robots could cooperate to solve a complex problem or perform the assignment in a more fault-tolerant, reliable, faster, or highly cost-effective manner than a single complex robot with all the capabilities [1]. In comparison to single-robot systems, multi-robotic systems could offer various advantages such as distributed sensing, distributed action because of acting in different places, improved performance through using parallelism, and fault tolerance due to the redundancy of the system [2]. However, the simplicity of these systems has raised other challenges, such as the higher probability of collisions and uncertainty about other robots’ intentions during the cooperation [2].

### 1.1. Swarm Robotics

Swarm robotics is an area of research in the field of artificial life and multi-robot systems, which aims to coordinate multiple and relatively simple robots in a distributed manner [3]. In robot swarms, robots’ interactions with other robots and the environment could lead to the emergence of desired collective behaviours for specific applications [4]. Swarm robotics is inspired by biological systems, notably social insects. Each individual in a natural swarm may show simple and single abilities; however, the whole swarm can exhibit complex behaviours, such as migrations of a flock of birds, schools of fishes, and foraging behaviour of bee or ant colonies [5]. It is indicated that individuals do not require sophisticated knowledge to perform these complex behaviours [6]. Specifically, local communication and information transmission among the individuals of a swam are believed to be responsible for the emergence of these complex behaviours [5]. The individuals of social insects have no knowledge of global colony or task status. In fact, in a swarm, knowledge is distributed throughout all agents, where a sole individual cannot accomplish a task without help from the rest of the swarm. Thus, individuals cooperate and interact locally to solve complex tasks. These characteristics contribute to the three principal advantages of natural swarms, which are desirable in multi-robotic systems: *scalability*, swarms of robots should be able to work either in small groups or in groups of thousands; *adaptability*, a swarm must be capable of creating multiple solutions for different types of tasks, and alter each robot’s role according to the situation; and *robustness*, a swarm of robots must still work, even when some individuals fail or disturbances occur in the environment [4]. These characteristics can be valuable when applied to artificial systems, leading to more robust engineering designs.

Robotic swarms have been deployed in the past decades in various areas of applications, including mobile sensor networking [7], odour localization [8], and search and rescue [9]. However, developing large robot swarms that coordinate and cooperate to perform a task or solve a problem remains a challenge [10].

### 1.2. Aggregation

Research in swarm robotics is typically focused on cooperative and collective behaviours among living things. Aggregation, foraging, coordinated movement, and task allocation are examples of these behaviours. However, among these collective behaviours, the aggregation behaviour plays a fundamental and often pre-conditional role [11,12]. Most studies on aggregation behaviour in swarm robotics have been inspired by the observable aggregation behaviour in nature [13,14]. Aggregation behaviour is one of the most frequent behaviours observed among many living creatures, such as birds, fish, insects, and mammals [15]. Different species live in aggregations for different reasons, such as anti-predator reactions, construction of a nest, etc.

Self-organizing and cue-based aggregation are two types of aggregation behaviours that have been observed in nature [16]. Cue-based aggregation usually relies on environmental cues such as temperature, light, or humidity for aggregation. In contrast, self-organized aggregation depends on emergent cooperative decision-making among individuals without relying on specific external cues [16]. The behaviour of aggregation in swarm robotics is similarly categorized into two distinct types, mirroring the classification observed in living organisms: cue-based [17,18] and self-organizing [19,20]. Natural phenomena such as bird flocks, mammal herds, and fish schools are interesting examples of such behaviours, offering effective strategies for coordinating numerous autonomous agents when only local information is available [5].

### 1.3. Reynolds’ Flocking Rules

In biology, flocking behaviours in natural systems have been investigated for a long time (e.g., see [21]). Reynolds [22] developed an algorithm that simulates the flocking behaviours of birds and created the artificial life program Boids, demonstrating that effective flocking can be achieved in an artificial swarm. In this work, simulated entities use the position and velocity data of nearby neighbours, along with their movement patterns, to collaboratively determine their subsequent actions based on defined behaviours. Reynolds proposed three heuristic rules for successful flocking behaviour, which are as follows:

- **Separation:** Steer to avoid colliding with nearby neighbours.
- **Alignment:** Steer toward the average heading of nearby neighbours.
- **Cohesion:** Steer toward the average position of neighbours.

Reynolds [22] defined flocking behaviour as “polarized, non-colliding, aggregate motion”. While both aggregation and flocking involve the collective movement of individuals, aggregation tends to focus on individuals coming together without strict coordination, while flocking involves coordinated movement patterns with alignment, cohesion, and separation behaviours. According to these rules, each entity moves toward the centre of mass of its neighbours, avoids collisions, and aligns its orientation with the average orientation of its neighbours.

This study introduces a method using Reynolds’s rules to aggregate robots scattered in an arena into a single group, addressing integration challenges such as collision among various groups of robots moving in the same direction. To improve the aggregation behaviour, we proposed using the K-means algorithm to identify clusters of agents. This simple approach can handle clustering a large number of individual data points and might, therefore, be useful for a swarm robotics system with large numbers of robots. The performance of the developed system was evaluated under various environments and conditions, such as various obstacles, agent failure, different numbers of robots, and arena sizes.

## 2. Background and Related Works

Swarm robotics is the practice of achieving a goal through a large number of simple robots. This implies that the swarm must be aggregated for cooperation in order to achieve the goal. Various methodologies, such as *probabilistic methods*, *evolutionary methods*, *artificial physics algorithms*, and *control algorithms*, have been suggested to tackle the challenge of self-organized aggregation in swarm robotics.

In probabilistic methods, aggregation behaviour is achieved using probabilistic finite state machines (PFSMs) inspired by the aggregation behaviour observed in natural systems, such as cockroaches and honey bees (e.g., see [23]). According to this model, the likelihood of a robot joining or leaving clusters depends on the sizes of the clusters. A greater probability is assigned for a robot to join a larger cluster, while a smaller probability is designated for leaving a smaller cluster (e.g., see [23,24]). Cambier et al. [25] integrated the PFSM model with naming game models [26] and observed a significant enhancement of aggregation capabilities.  Firat et al. [27] studied the aggregation of swarm robots at specific locations within a predefined scenario controlled by a Finite State Machine (FSM). In their research, the authors used informed robots, which are members of a swarm programmed to stop only at experimenter-defined locations for aggregation [27]. In some works, artificial evolution techniques have been employed to simulate self-organized aggregation models. In this method, the robot is typically controlled by evolutionary algorithms or neural networks, which map sensory inputs into output commands for the robot’s actuators (e.g., see [28,29]).

In artificial physics algorithms, the process of aggregation is commonly realized by applying artificial laws of physics to simulate the interactions between robots. For example,  Gasparri et al. [30] attained effective aggregation results by employing attractive and repulsive artificial forces among a swarm of robots. In another study, Güzel et al. [31] proposed a decentralized strategy for multi-agent systems to facilitate collective and collaborative behaviours. This framework introduces a new clustering behaviour based on a vision-based goal detection algorithm, allowing swarm agents to autonomously agree to join a group and assign a leader to each group in a decentralized and local manner [31]. In this work, the leader of each group uses a vision-based goal detection algorithm. Once the leader begins moving, each member follows the leader and moves in the same direction, ensuring the maintenance of the desired formation pattern [31]. Another work investigated the collective aggregation behaviour within a swarm of simulated versions of foot-bot robots, which are differential mobile robots with two wheels, equipped with a set of actuators and sensors [32]. This method models inter-robot interactions through virtual viscoelastic forces. Foot-bots only interacted with their K-nearest neighbours (KNNs) instead of all neighbours within their field of vision. Each robot selected its KNNs solely based on distance constraints [32]. The concept is inspired by the observation that individuals in the biological systems interact only with their closest neighbours (around six or seven) rather than with all the available neighbours in their field of view [33,34]. Later, an extension of that approach, known as Distance-Weighted K-Nearest Neighbors (DW-KNNs), was implemented to include robot density as an additional factor to improve the performance of swarm aggregation [35,36]. In this technique, distances to neighbours are adjusted based on density-based estimation values obtained from the neighbours. Subsequently, the robots are virtually drawn toward their K-nearest neighbours with the smallest weighted distances [35,36].

**Figure 1:**
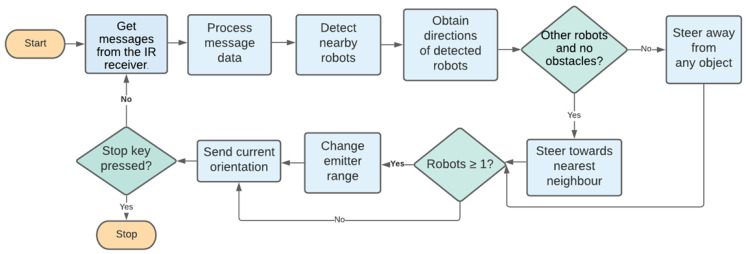
Reynolds aggregation algorithm state diagram for a single robot [39].

In studies for control algorithms, Mısır et al. [37] introduced a novel fuzzy logic-based self-organizing aggregation technique. Unlike conventional self-organizing aggregation methods in swarm robotics, the approach involved the use of fuzzy logic controllers to assess limited sensor data [37]. Misir and Gökrem [38] also introduced an aggregation method for swarm robotics inspired by flocking behaviour. Their method focuses on self-organizing behaviour in a swarm of non-holonomic robots, which can detect and communicate with neighbouring robots within a predefined sensing radius. Each robot is equipped with eight obstacle detection lidars and a compass sensor to share and measure the heading angles of neighbouring robots. When the robots detect neighbours within a radius of 5, 6, or 7 units and at a distance of 3.5 units, they rotate based on the average value of the detected angles. If the distance to neighbouring robots is between 1 and 3.5 units, the robots exhibit flocking behaviour. Systematic experiments were conducted using the Mobile Robotics Simulation Toolbox in MATLAB to test the aggregation method with varying arena sizes, robot numbers, and detection distances [38].  Fraser [39] presented a bio-inspired aggregation method based on Reynolds’ flocking rules to achieve the self-organizing aggregation behaviour in a swarm of robots through local interactions. In this algorithm, the flocking behaviour is based on the information that each individual e-puck robot [40] has about its neighbours. The flocking behaviour was simulated using Webots robotics simulation software 7.0.1 [41]. To obtain data from the environment, each e-puck robot uses its IR sensors, which can function as proximity and light sensors. To enable communication between robots, one emitter and receiver node of the Webots have been used for each e-puck robot. In Webots simulation software [41], a node is a fundamental element representing objects or components in the 3D simulation, such as robots, sensors, or environmental features. These nodes are organized in a hierarchical structure called the scene tree, defining specific properties and behaviours. To enable the robot to receive a localized bearing of the emitter without needing centralized control, a compass module was added to each e-puck in order to simulate the “Range and Bearing Turret” of real e-pucks. Therefore, each robot can communicate with each other. This algorithm contains a message protocol to receive and process the information of each message from another robot. Each communicated message contains the number of e-puck robots in each robot neighbourhood detected using IR sensors, the signal strength, which represents the distance of the message signal, and the message signal direction. Data on angle and Euclidean distance allow the e-puck robot to determine the message’s origin. To implement *heading alignment*, each robot obtains directions from the detected robots and calculates the target heading alignment vector. If there are other robots and no obstacles, then the robot will steer toward the direction of its nearest e-puck robot and change its emitter range to send its current orientation. Otherwise, the e-puck robot will perform obstacle avoidance. To enforce the *cohesion rule*, the new emitter range is calculated regularly to avoid individuals being too close or too far apart. Finally, to implement the *separation rule*, each robot’s IR sensors detect objects and other robots. Obstacle avoidance will be achieved through polling the proximity sensors. The data on the sensor’s position will assist in locating the obstacle. The procedure of this algorithm is shown in Figure 1.

As indicated in [39], the main limitation of using Reynolds’ rules for aggregation behaviour is that increasing the arena size results in longer execution times and affects the scalability of the swarm due to poor communication between the agents.

## 3. Materials and Methods

In this paper, we developed a biologically inspired robot controller based on Reynolds’ rules to coordinate the movements of multiple e-puck robots during the aggregation process. To address the scalability issues of the flocking-based aggregation approaches, we used a K-means algorithm to identify clusters of agents. To evaluate the performance of our proposed method, we compared it with an available robot controller based on the work of [39]. We will refer to it as the “Reynolds” approach controller in the following. This section details the design and implementation of our proposed algorithm and the approaches employed to address the challenges in multi-agent interactions.

### 3.1. Simulation World

In this study, Webots simulation software [41] has been used for 3D simulation and implementation of the experiments. Webots provides a free and open-source professional simulation environment for both virtual and real mobile robots. This software supports different programming languages for robot controllers, where the behaviour of the robots is defined. For this project, all the robot controllers have been written in the C and C++ programming languages, and the source code is available at GitHub (Link to the source code at https://github.com/Sami-Ra/SwarmRobotics (accessed on 15 October 2024)).

### 3.2. E-Puck Robotic Platform

**Figure 2:**
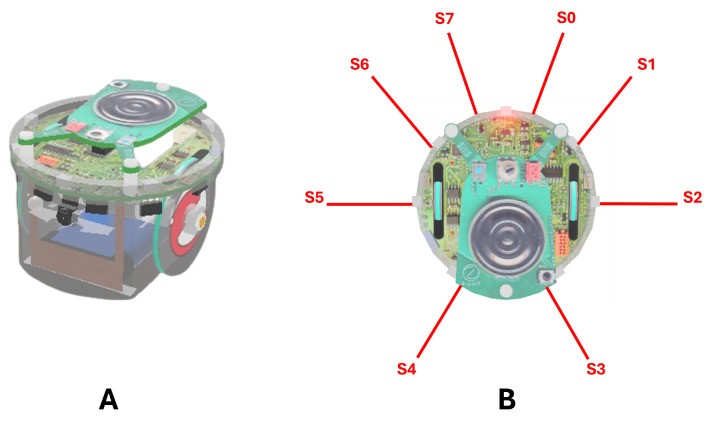
(**A**) e-puck mobile robot, (**B**) e-puck sensor positions: S0–S7 indicate the positions of the IR sensors.

We used an open-source robotic platform, the e-puck mobile robot [40]. The e-puck is 7.4 cm in diameter and has two wheels to move. The e-puck’s flexibility, low cost, and small size make it well-suited for a hardware-based research approach. In addition, the e-puck is equipped with different useful sensors and actuators such as 8 Infra-red (IR) sensors for detecting the proximity of obstacles, light sensors, the 3D accelerometer, camera, etc., (see Figure 2). The e-puck’s integration with Webots software makes it easy to program, cross-compile, simulate, and control the robot.

### 3.3. Reynolds_K-Means Algorithm

This study mainly aims to design a robot controller that could drive the swarm of *N* e-puck mobile robots to aggregate somewhere within an arena without any predetermined aggregation zones. Thus, to achieve the desired aggregation behaviour, it is necessary to design an aggregation control model to determine the proper input control parameters for e-puck robots.

The Reynolds algorithm has one major drawback, which is that the three defined rules (i.e., cohesion, alignment, and separation) must be calculated by using an entity that knows the position and orientation of each robot as it is an artificial life principle to use only information available from the agent’s own perspective (as much as possible) [42]. Therefore, in this study, we developed two robot controllers: (1) “*e-puck controller*” that is the local controller of each robot and is used to implement the Reynolds’ separation rule and adjust the orientation, speed, etc., for each robot based on local information, and (2) “*my_Supervisor controller*” where a supervisor node is defined to receive the position of each robot through messages. This controller implements the aggregation and cohesion rules of the Reynolds strategy for robots using the K-means algorithm to determine new locations and then sends these updated coordinates back to the robots. Hence, the robots that are close to each other will aggregate together as one cluster. The idea is inspired by the observation that in biological systems, individuals tend to interact primarily with their nearby neighbours rather than with all other individuals [33,34]. By forming local clusters, we could simulate natural systems’ distributed, gradual aggregation. This approach could also enhance the scalability of simulations of flocking-based aggregation behaviour in larger arenas, as smaller groups can first aggregate locally before converging toward a global center. Thus, it could offer practical benefits for swarm coordination in dynamic, real-world environments.

#### 3.3.1. Flocking Behaviour

We developed a robot controller to coordinate the movements of multiple robots in small groups using Reynolds’ flocking rules when these robots are moving to aggregate and form a single final group. To obtain data from the environment, each e-puck robot uses its eight IR sensors as proximity sensors. These sensors allow the e-puck robot to observe its immediate environment and react to it based on the processed sensory inputs. E-pucks also contain a variety of wired and wireless communication channels, primarily used in communication with a host computer (supervisor node). This technology allows for the control of the robot using wireless technology and provides full remote access to the sensory data [40]. To establish communication between the supervisor node and e-puck robots in Webots, we integrated emitter and receiver nodes into both the supervisor node and the e-puck robots. In this work, the emitter and receiver nodes are used to model radio emitters and receivers. The emitter node is responsible for transmitting messages, while the receiver node captures and processes these messages. For example, in the supervisor controller, the emitter sends control commands to the e-puck robots, and each e-puck robot’s controller receives and processes these commands via its receiver. Likewise, the robots can transmit data back to the supervisor node using their own emitters, creating a two-way, real-time communication channel throughout the simulation. In this study, a compass module is added to each robot that is used for heading alignment. The compass module provides data that the robot can use to determine its heading in the environment. The achieved data from the compass module are in the format of three-dimensional vectors in the direction of the north point in the simulation.

To implement a flocking-based aggregation behaviour, we use a two-step strategy: (i) Find the local groups of robots in close proximity to enforce the rule of aggregation. (ii) To promote the rule of cohesion, try to centralize these local groups. For achieving the first step, we use the K-means clustering algorithm [43]. After that, the heading of the robots in each local group (each cluster) is adjusted toward the global center of all groups.

#### 3.3.2. Cohesion Performance Metric

To create an evaluation metric for measuring cohesion, first, we use the following relation to compute the sum of distances between each robot and the global centre of the mass:

(1) $$D\left[t\right]=\frac{1}{N}\sum_{i=1}^{N}d\left(CM\left[t\right],robot\left[i\right]\right),$$

where d(.,.) represents Euclidean distance between two positions, and CM[t] and $robot_{i}\left[t\right]$ are the global centre of the mass and the position of the robot *i* at time *t*, respectively. After that, we obtain the cohesion measure at time *t* from the equation below:

(2) $$C\left[t\right]=\frac{1}{1+D[t]}.$$

It can be seen that 0⩽C[t]⩽1. The value near 0 represents weak cohesion and the value near 1 represents strong cohesion for each algorithm at time *t*.

#### 3.3.3. K-Means

K-means clustering is a popular unsupervised machine learning algorithm [43]. In machine learning, several data points found to be similar can be aggregated together as a cluster. There are several similarity measures, such as correlation-based distance and Euclidean-based distance, that can be used to identify homogeneous subgroups in data. We minimize the following cost function for implementing the K-means clustering algorithm [44].

(3) $$J=\sum_{n=1}^{N}\sum_{k=1}^{K}r_{nk}{∥x_{n}-\mu_{k}∥}^{2}$$

where *N* is the number of data points, ${\left\{x_{i}\right\}}_{i=1}^{N}$, the number $r_{nk}\in \left\{0,1\right\}$, k=1,...,K is used to determine if data point $x_{n}$ is assigned to cluster *k*, ($r_{nk}=1$), or not ($r_{nj}=0$ for j≠k), and $\mu_{k}$ represents the centres of cluster *k*th. The goal is to find $r_{nk}$ and $\mu_{k}$ values such that *J* is minimized.

In this study, first, the K-means algorithm selects *K* cluster centres randomly and distributes robots among clusters using the Euclidean distance (similarity measure) of each robot from the centre of the cluster. Thus, the robots that are close to each other will aggregate together as one cluster. The idea is based on the observation that in biological systems, individuals typically interact with only their immediate neighbours rather than engaging with all potential neighbours [33,34]. Then, the algorithm computes new cluster centres based on the current robots’ positions in the cluster. This procedure is repeated until all the robots are clustered. By executing the K-means algorithm, the robots in each local group will be directed toward the centre of their group (swarming behaviour) (see Algorithm 1).
**Algorithm 1:** Alignment Algorithm**Input:**    *K*: Number of Clusters1:currentCoordinate←getCurrentCoordinate()2:Use *K -means* clustering algorithm to find:3:    Membership of each robot to a certain cluster4:    clusterCenter of each cluster5:For each robot *i*:6:    sendMessage(roboti,clusterCenter[i])

The supervisor controller then computes the global centre of the *K* cluster centres. After reaching a relative maximum cohesion of the swarm of robots, calculated based on Equations (1) and (2) for measuring cohesion performance (see Section 3.3.2), the alignment algorithm is executed once more to direct all the robots to the coordinates of this global centre. To achieve *heading alignment*, the supervisor controller sends a message to each e-puck robot with the coordinates of the global centre of all of the groups. Upon receiving the message, robots in each cluster steer toward the direction of the global centre. The e-puck robots move towards the global centre until they achieve a relative maximum cohesion within the swarm (see Algorithm 2). At this stage, the flocking behaviour of each group and the cohesion rule will be satisfied (see Algorithm 3).
**Algorithm 2:** Receiving Message in Each Robot**Event:**    Receiving a Message1:newDestination←ReadInputMessage()2:currentCoordinate←getCurrentCoordinate()3:**IF **(currentCoordinate≠newDestination):4:    **For** each robot *i*:5:        compass←getCompassValue()6:        angelToDest←calcAng(newDestination)7:    changeRobotHeading()8:    goToDestinationPoint()

**Algorithm 3:** Cohesion Algorithm

**Inputs:**

    *K*: Number of Clusters
    clusterCenter: Array of cluster centres
1:

globalCenter←0

2:For each cluster *i*:3:    globalCenter←globalCenter+clusterCenter[i]4:

globalCenter←globalCenter/K

5:**For** each robot *i*:6:    sendMessage(roboti,globalCenter)


#### 3.3.4. Separation Algorithm:

Another important factor in flocking behaviour is obstacle avoidance. The IR sensors of each robot are used to control the proximity between the robots in a swarm and avoid obstacles that might be in the way. A threshold is defined for each IR sensor on the robot. If the value of the IR sensor is above the threshold, its information will be saved as proximity information. The data on the sensor’s position will assist in locating the obstacle. In our case, if the IR sensor’s value is more than 90, we set the corresponding value in a Boolean array, ‘Obs’, to true; otherwise, we set it to false. The velocity of the left and right wheels will be adjusted accordingly to avoid obstacles (see Algorithm 4).
**Algorithm 4:** Separation Algorithm**Inputs:**     Obs[0..7] 1:letleft_obs←Obs[5]ORObs[6]ORObs[7]; 2:letright_obs←Obs[0]ORObs[1]ORObs[2]; 3:letback_obs←Obs[3]ORObs[4]; 4:letfront_obs←Obs[0]ORObs[7]; 5:**IF**(front_obsANDleft_obsORfront_obs) 6:    GoBackward 7:    TurnRight 8:**Else If **front_obsANDright_obs 9:    GoBackward10:    TurnLeft11:**Else If **back_obs12:    MoveForward13:**End If**

## 4. Research Questions

We have presented the research questions below.

1. Does the developed swarm system exhibit flocking behaviour using robots’ information?
2. How quickly do robots aggregate to form a flocking behaviour using robots’ information?
3. Is the system capable of exhibiting flocking behaviour in the presence of various obstacles in the arena?
4. Is the swarm of robots able to continue flocking behaviour in the event of a failure of one or more robots?
5. Is the developed swarm system scalable?

## 5. Experiments Results and Discussion

In this paper, five experiments were implemented to answer the research questions. To simulate a swarm robotics system, e-puck robots were randomly distributed and oriented towards arbitrary directions in a bounded arena of size 3 m × 3 m with 1 cm wall height.

### 5.1. Experiment 1: Exhibiting Flocking Behaviour

**Figure 3:**
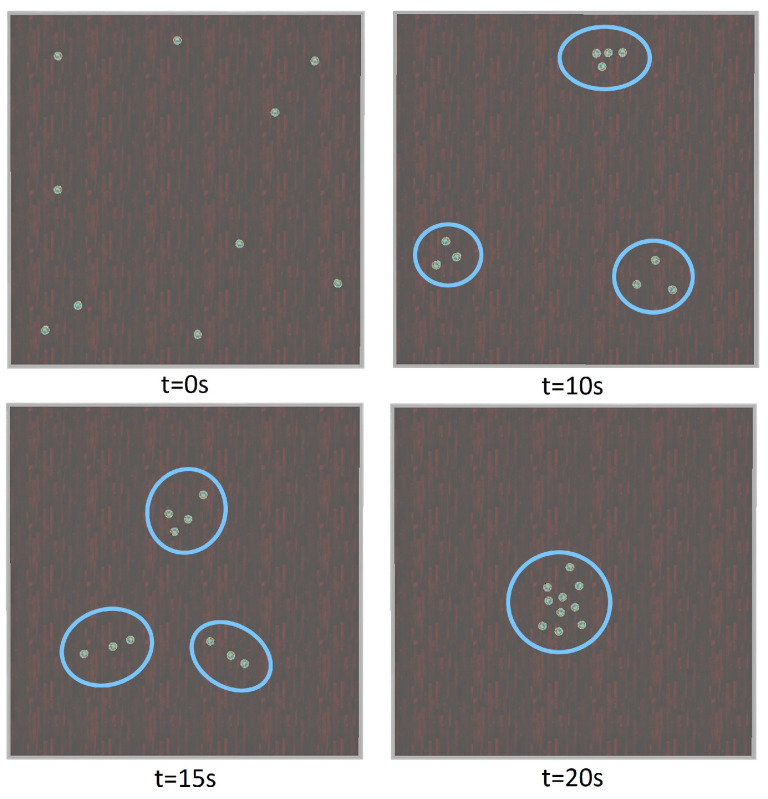
Aggregation behaviour over time (blue circles show clusters of e-puck robots at time *t*).

To answer the first research question, we investigated whether all robots move as a group and exhibit flocking behaviour or not. This was examined by executing the simulation program and observing the robots’ behaviours. In this experiment, robots were scattered randomly in the arena, and by running the algorithm, we expected them to aggregate and move together in a swarm. Results are shown in Figure 3.

At the beginning of the simulation, all the robots were scattered randomly in the arena (*t* = 0 s); after executing the algorithm, the robots were partitioned into *K* = 3 clusters and began to form three cohesive clusters (*t* = 10 s). Then, all the robots in each cluster adapted their heading direction (alignment algorithm) toward the centre of all three clusters’ centres, and robots in each cluster moved together in a swarm (*t* = 15 s) to form a bigger swarm of robots. By (*t* = 20 s), all the robots had aggregated and formed a uniform cluster (see Figure 3).

**Figure 4:**
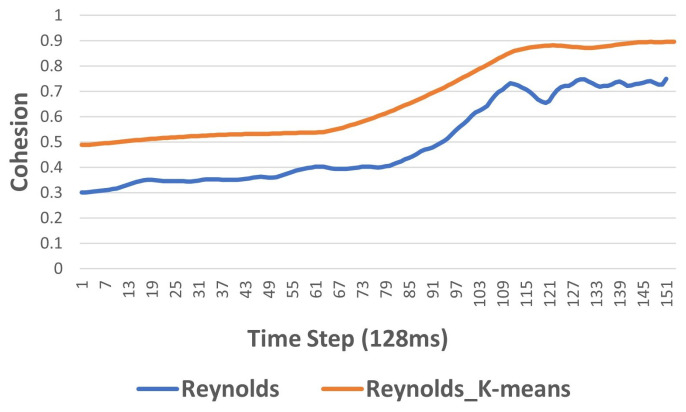
Evaluating cohesion performance of Reynolds algorithm and Reynolds_K-means algorithm.

As illustrated in Figure 4, comparing the cohesion performance measure of the Reynolds algorithm [39] and our proposed algorithm (Reynolds_K-means algorithm) shows that our algorithm outperformed the Reynolds algorithm [39] in terms of both the speed of convergence and quality of cohesion measure (the closer the value is to 1, the stronger the cohesion for each algorithm at time *t*).

### 5.2. Experiment 2: Aggregation Time

To answer the second research question, e-puck robots were randomly scattered in the arena, and the average time of multiple simulation runs was calculated. In this experiment, we ensured consistency by using the same parameters and number of robots as in [39] for a direct comparison of results. As can be seen in Table 1, our proposed method was faster than the Reynolds approach with the average aggregation time of 23.52 seconds, and also, there is a smaller variance between the run times. It can be argued that defining a higher-level controller (supervisor node) that directs the behaviour of each e-puck robot can improve the aggregation time. Additionally, the results of our proposed method demonstrated the robustness of the aggregation after the aggregation process was completed, as the robots maintained their cohesion after aggregation.

### 5.3. Experiment 3: Exhibiting Flocking Behaviour in the Presence of Obstacles

**Figure 5:**
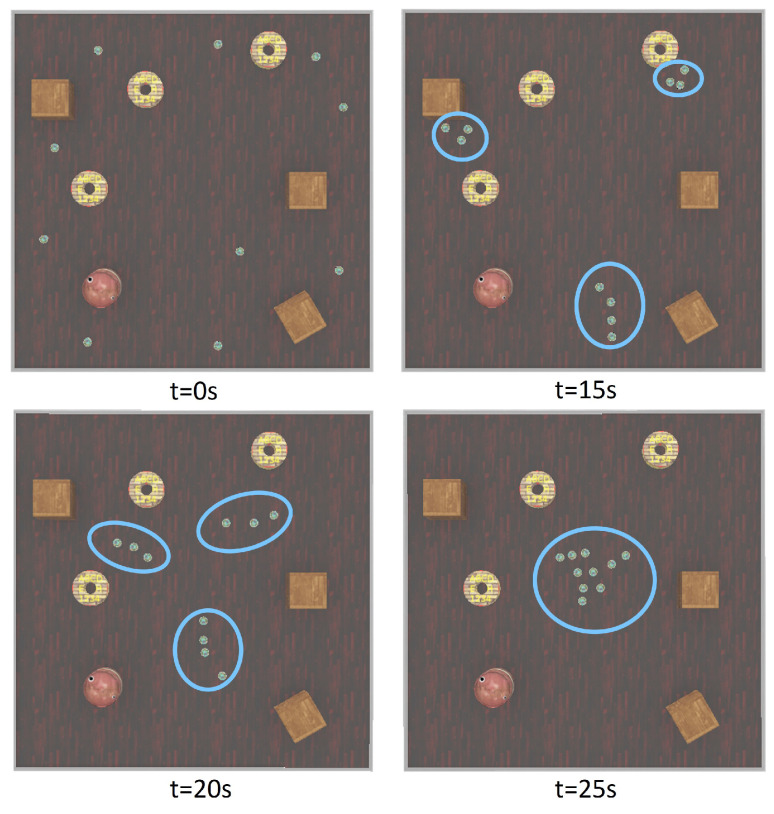
Exhibiting aggregation behaviour in the presence of various obstacles (blue circles show clusters of e-puck robots at time *t*).

To answer the third research question, we designed an experiment in which the robots were scattered randomly in the arena, and different obstacles of various sizes and shapes were presented in the arena. In this way, we tested our proposed obstacle avoidance strategy, which was built into the Reynolds strategy. As can be seen in Figure 5, e-puck robots were able to successfully aggregate and exhibit flocking behaviour in the presence of various obstacles. However, a slight increase in aggregation time is observed.

### 5.4. Experiment 4: Effect of Faulty Robots

**Figure 6:**
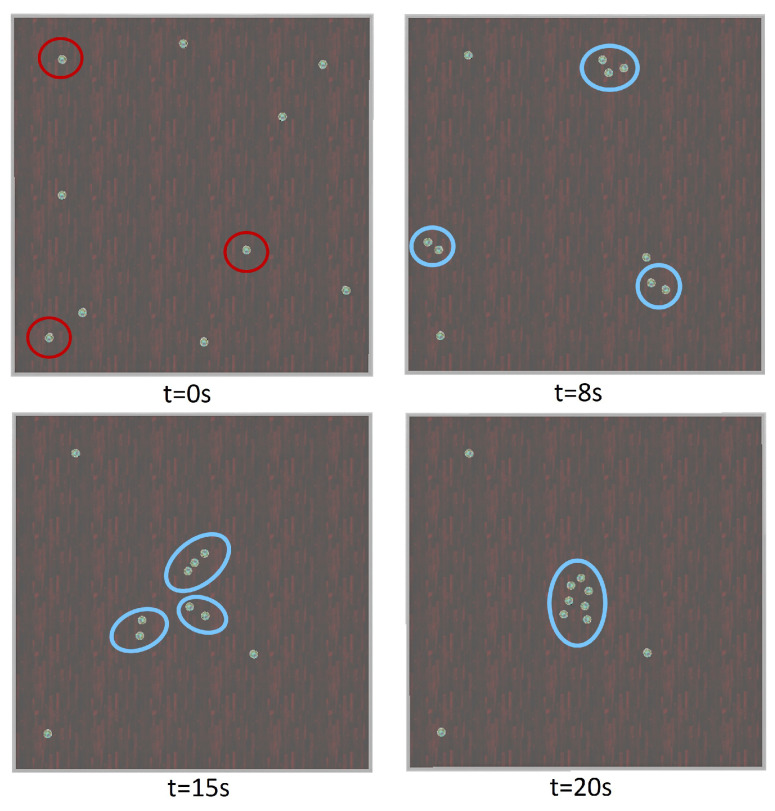
Failure test (the faulty robots are shown in red circles and blue circles indicate clusters of e-puck robots at time *t*).

To answer the fourth research question, all the robots were scattered randomly in the arena. Then, three robots were powered down to simulate failure at (*t* = 0 s). Since the inactive robots do not send any signals to the supervisor node, it is expected that the system continues exhibiting flocking behaviour and treats inactive robots as if they are some obstacles in the environment that the system needs to avoid. Results are shown in Figure 6. In this figure, the inactive robots are shown in red circles. By running the simulation, the robots are partitioned into *K* = 3 clusters (blue circles) and begin to form three cohesive clusters (*t* = 8 s). The robots ignore the three inoperable robots and aggregate at the centre of their clusters. Then, all the robots in each cluster adjust their heading direction, using the alignment algorithm, toward the centre of all three clusters’ centres and move together in a swarm (*t* = 15 s) to form a bigger swarm of robots at (*t* = 20 s). According to the results of this experiment, our proposed method still works even when some robots fail, which demonstrates the robustness of the algorithm.

### 5.5. Experiment 5: Scalability Evaluation

**Figure 7:**
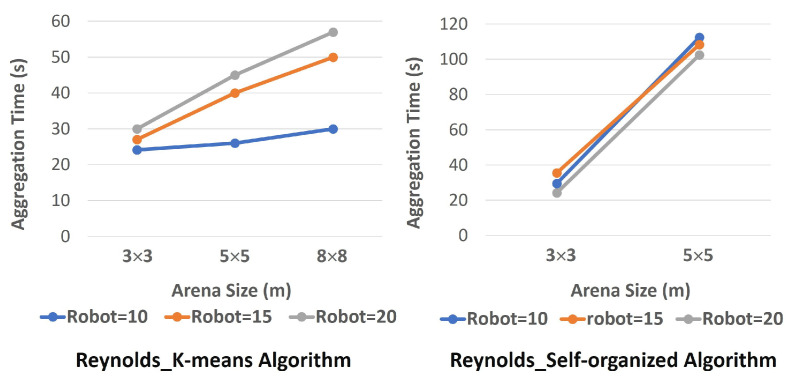
Scalability evaluation.

The purpose of the fifth research question is to measure the scalability of the system, which is one of the key characteristics of a robot swarm [4]. To answer this research question, we tested different swarm sizes by introducing more robots to the swarm and observing the system’s flocking behaviour. For instance, starting with 10 robots, we increased the number of robots to 15 and 20 and conducted simulations. Additionally, we varied the area sizes from 3 m × 3 m to 5 m × 5 m and 8 m × 8 m. As can be seen in Figure 7, increasing the number of robots and arena size does not affect the performance of our proposed method and shows that the proposed algorithm is scalable. The reason could be the scalability of the K-means algorithm, which can handle a large number of individual data points. Thus, the system could adapt to such a change and continue its flocking behaviour even with the added workload of adding extra agents. However, the performance of the Reynolds algorithm decreased, and for arena size 8 m × 8 m, the robots were not able to aggregate and show flocking and swarming behaviour. The reason could be the fact that the e-puck robots in this approach communicate locally, and by increasing the arena size, the robots cannot receive the signals they exchange.

In a nutshell, while our proposed method is scalable in terms of both increasing the number of robots as well as the size of the arena, the Reynolds algorithm is scalable only in terms of increasing the number of robots, as can be seen in Figure 7.

**Figure 8:**
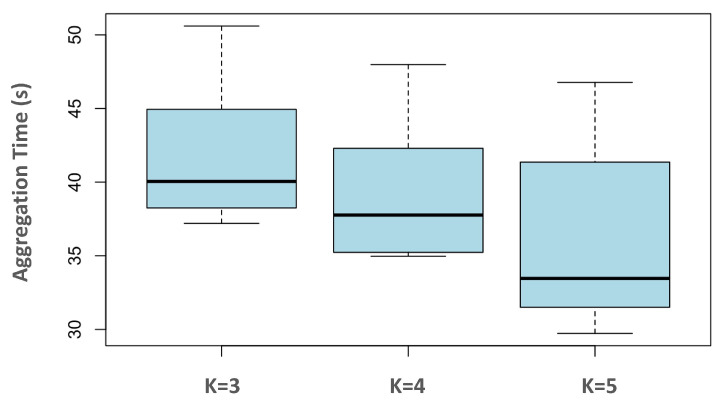
Aggregation time for different values of *K*.

In addition, we conducted a series of experiments using the Reynolds_K-means algorithm with varying values of *K* (i.e., 3, 4, and 5) to assess the algorithm’s performance under different clustering configurations. Each experiment was run 50 times to ensure robustness. The experimental setup involved 30 e-puck robots operating in an arena of size 5 m × 5 m. The aggregation time results for different numbers of clusters are presented in Figure 8.

## 6. Conclusions and Future Work

Aggregation represents a fundamental aspect of flock behaviour in most biological systems and is crucial for many robotic systems. It serves as a prerequisite for various collaborative activities and allows robot swarms to perform complex tasks, such as information exchange, self-assembly, collective movement, etc. In this work, we developed a biologically inspired robot controller to coordinate the movements of multiple robots during the aggregation process based on Reynolds’ flocking behaviour.

Reynolds demonstrated that three simple rules of (i) cohesion, (ii) separation, and (iii) alignment are sufficient to achieve flocking behaviour. Robot controllers inspired by Reynolds’ local rules use local information regarding immediate neighbours. However, this approach has its limitations. For instance, increasing the arena size affects the scalability of the swarm of robots due to poor communication between the agents. Thus, we decided to add a second controller (i.e., supervisor controller) that also considers the global behaviour of the entire swarm rather than only local interactions among neighbouring agents.

Previous research has also explored the implementation of flocking behaviour in embodied agents based on the global application of Reynolds’ rules, which showed improved global flocking behaviour in comparison to the local application of Reynolds’ rules [45].

In this work, we proposed using a K-means algorithm that uses distance metrics with Reynolds’ rules to improve the flocking and aggregation behaviour among e-puck mobile robots. The aim was to design a robot controller that could drive the swarm of e-puck robots randomly distributed in an arena to aggregate somewhere within an arena without any predetermined aggregation zones. Using this robot controller, we simulated aggregation behaviour among multiple robots.

To the best of our knowledge, this is the first study to use the K-means algorithm in this field. Since K-means is an efficient method for handling a large number of individual data points, it could be an effective approach and provide a more robust controller in swarm robotics, where scalability, robustness, and adaptability are key factors [12].

The performance of our proposed method was examined via five different experimental analyses. Various environmental factors and conditions, such as agent failure, obstacles, arena size, and flock merging, have been considered in each experiment to measure the system’s performance. Swarms of robots are often faced with these types of environmental challenges, and our proposed algorithm’s capability to cope effectively with these dynamic environmental conditions could demonstrate a proof of concept of the applicability of our proposed method in swarm robotics. The results of the experiments showed that the proposed algorithm is robust and can easily deal with agent failures. Furthermore, the Reynolds_K-Means algorithm is scalable in terms of increasing both arena size and the number of robots. However, one limitation of this method is that we need to define the number of clusters of the robots before executing the algorithm.

In this study, we employed K-means, which uses distance metrics to improve aggregation behaviour. While some studies used the distance between robot neighbours as the key factor to implement aggregation behaviour in a swarm of robots [32], some other recent studies have proposed the incorporation of agent density as a supplementary factor to improve the performance of the swarm aggregation [11,35,36]. Thus, this work can be extended by applying density-based approaches to determine the number of clusters. However, this may increase the complexity of the algorithm in comparison to the K-means, which is a simple and fast algorithm.

## Figures and Tables

**Table 1:** Average time to aggregation.

	Average Time (s)	
Run	*Reynolds_K-Means*	*Reynolds*
1	22.21	29.44
2	25.06	12.16
3	24.36	46.68
4	21.71	59.93
5	24.28	29.24
Average	23.52	35.49

## Data Availability

The original contributions presented in the study are included in the article, further inquiries can be directed to the corresponding author.
